# Coping styles predict responsiveness to cognitive behaviour therapy in psychosis

**DOI:** 10.1016/j.psychres.2010.12.029

**Published:** 2011-05-30

**Authors:** Preethi Premkumar, Emmanuelle R. Peters, Dominic Fannon, Anantha P. Anilkumar, Elizabeth Kuipers, Veena Kumari

**Affiliations:** a Department of Psychology, Institute of Psychiatry, King's College London, London, UK; b Division of Psychology, School of Social Sciences, Nottingham Trent University, Nottingham, NG1 4BU, UK; c NIHR Biomedical Research Centre for Mental Health, South London and Maudsley NHS Foundation Trust, London, London, UK; d South London and Maudsley NHS Foundation Trust, London, London, UK

**Keywords:** Coping skills, Cognitive behaviour therapy, Psychosis

## Abstract

The study aimed to determine the clinical and neuropsychological predictors of responsiveness to cognitive behavioural therapy for psychosis (CBTp). Sixty patients with schizophrenia or schizoaffective disorder and 25 healthy individuals took part in the study. Thirty patients (25 protocol completers) received CBTp in addition to standard care (SC); 30 patients (18 protocol completers) received SC only. All patients were assessed on symptoms using the Positive and Negative Syndrome Scale (PANSS) and clinical and neuropsychological function before and after CBTp. Symptoms and self-esteem improved to a greater extent in the CBTp + SC than SC control group. Greater pre-therapy coping ability and the self-reflectiveness dimension of cognitive insight at baseline predicted improvement in symptoms in the CBTp + SC group, but not the SC control group, explaining up to 21% of the variance in symptom improvement. Pre-therapy neuropsychological function, duration of illness, clinical insight and gender did not predict CBTp responsiveness. Being able to have a range of coping strategies and reflect on one's experiences while refraining from overconfidence in one's interpretations before therapy is conducive to better CBTp responsiveness.

## 1 Introduction

The extent to which neuropsychological and clinical factors determine responsiveness to cognitive behavioural therapy (CBT) for individuals with a psychotic disorder remains unclear. Seven randomised controlled trials (RCTs) have examined the clinical predictors of CBT for psychosis (CBTp) (Tarrier et al., 1993; Garety et al., 1997; Tarrier et al., 1998; Naeem et al., 2008; Brabban et al., 2009; Emmerson et al., 2009; Perivoliotis et al., 2010) (see Supplementary data for a summary of the studies), but have yielded somewhat contradictory findings.

Tarrier et al. (1993) found that greater pre-therapy positive symptom severity predicted greater positive symptom improvement following CBTp. In contrast, Naeem et al. (2008) combined the results of two relatively large RCTs of CBTp versus treatment as usual in the Insight trial (*N* = 422) and versus befriending in the London Newcastle trial (*N* = 90), and in the Insight trial, reported that lower pre-therapy general psychopathology predicted the greater likelihood of a good treatment outcome in the CBTp group, defined as a 25% improvement in general psychopathology, but not positive symptoms. Tarrier et al. (1998) found that the likelihood of a 50% improvement in positive symptoms was predicted by a shorter duration of illness in those receiving CBTp, but not in those receiving routine care or supportive counselling, while Garety et al. (1997) did not find relationships between length of illness or age of onset and outcome, but observed that having a greater number of admissions in the previous five years was related to subsequent improvement in positive symptoms. Brabban et al. (2009) reported that being female was associated with a greater likelihood of improvement in general psychopathology, while again Garety et al. (1997) did not.

There are, however, more consistent results showing that some aspects of insight may be related to good outcome in CBTp. Both Garety et al. (1997) and Brabban et al. (2009) showed that cognitive flexibility about delusions, i.e. acknowledging that another view of the delusion may be possible, and having lesser conviction in one's delusional beliefs, was associated with a greater likelihood of improvement. Naeem et al. (2008) found that better pre-therapy insight into ‘illness’ predicted good outcome in the Insight study. Emmerson et al. (2009) found that greater clinical insight [awareness that the patient has a mental illness or specific symptoms, awareness of need for treatment, and attribution of symptoms to mental illness, as assessed by the Birchwood Insight Scale (BIS, Birchwood et al., 1994)] at baseline predicted greater improvement in independent living skills following CBTp, while Perivoliotis et al. (2010) reported that greater cognitive insight (the patient's ability to reflect on his/her experiences and recognise that conclusions may be incorrect), in particular self-reflectiveness (Beck Cognitive Insight scale, BCI, Beck et al., 2004), was associated with an improvement in severity of delusions and auditory hallucinations. While the Garety et al. (1997) study did not find that clinical or illness insight was predictive of good outcome, insight into the social consequences of one's illness predicted improvements in the CBTp group.

Neuropsychological ability in the form of general intelligence or specific neuropsychological functions, such as memory, attention and executive function, may also play an important role in the therapeutic success of CBT. Neuropsychological impairment in individuals receiving therapy for alcohol dependence, for example, can impede acquisition of new coping behaviours (McCrady and Smith, 1986) or learning and retention of new material (Alterman and Hall, 1989). Three studies (Garety et al., 1997; Granholm et al., 2008; Penades et al., 2010) (see Appendix A for a summary of the studies) examined pre-therapy neuropsychological predictors of CBTp response in patients with a schizophrenia or schizoaffective disorder diagnosis, of which two studies (Garety et al., 1997; Penades et al., 2010) targeted symptom improvement, while Granholm et al. (2008) targeted dysfunctional performance beliefs that interfered with social functioning. Penades et al. (2010) reported that schizophrenia patients who showed a reliable improvement in symptoms had greater pre-therapy verbal memory than non-improvers. Neither Garety et al. (1997) nor Granholm et al. (2008) found that IQ or neuropsychological performance were predictive of good outcome. However, Garety et al. (1997) did not use a comprehensive neuropsychological battery, while CBT in the Granholm et al. (2008) study did not target symptom reduction.

One area which has not been investigated in CBTp outcome research is coping style. Coping enhancement has formed a part of some forms of CBTp (Tarrier et al., 1993; Tarrier et al., 1998; Andres et al., 2003) and been associated with better therapeutic outcome than standard care (SC) or supportive counselling. Patients' mastery of active, problem-focused coping strategies immediately after completion of therapy is reported to predict better psychopathological and social outcome 12–18 months after therapy (Andres et al., 2003). The successful acquisition of coping strategies during therapy may be dependent on an individual's pre-existing coping style, thereby influencing therapeutic outcome. No study, to our knowledge, has yet examined the role of pre-therapy coping styles in CBT responsiveness in psychosis.

The present study aimed to determine the pre-therapy clinical and neuropsychological predictors of CBTp responsiveness in patients with schizophrenia or schizoaffective disorder. Based on earlier findings and the literature about the role of coping in CBTp, we tentatively hypothesized that a shorter duration of illness, better cognitive insight, more active coping, greater verbal memory and cognitive flexibility at baseline would be associated with better CBTp responsiveness, as measured by significant clinical improvement on the Positive and Negative Syndrome Scale (PANSS, Kay et al., 1987). The study also examined the change in clinical status and neuropsychological function following CBTp. It was hypothesized that symptoms and cognitive insight would improve following CBTp, since improvement in symptoms was associated with greater post-therapy cognitive insight in two previous studies (Granholm et al., 2002; Perivoliotis et al., 2010).

## 2 Methods

### 2.1 Participants and design

Participants were 60 outpatients with a DSM-IV diagnosis of schizophrenia or schizoaffective disorder who were willing to receive CBTp in addition to their usual care. Twenty-five healthy participants were also included in order to characterize the patient group. The patient and healthy participant groups were matched on average for age and sex. Data on the improvement of symptoms in the CBTp + SC group compared to SC control group following CBTp have been reported in our recent reports on the functional MRI predictors (Kumari et al., 2009; Kumari et al., 2010) and structural MRI predictors of CBTp responsiveness (Premkumar et al., 2009). Thirty patients received CBTp + SC (25 protocol completers) and 30 patients received SC (18 protocol completers in this part of the study). There were more drop-outs in the SC control group than the CBTp + SC group (Chi-square = 4.022, *p* = 0.045). Of those recruited to the CBTp + SC group, five patients left the study early (one patient discontinued therapy and four patients withdrew consent). Of those recruited to the SC control group, 12 patients dropped out (five patients withdrew consent; five patients did not undergo baseline MRI and were not followed-up, one patient moved to another area and could not be contacted, and one patient was incarcerated during the period of his scheduled follow-up). The five CBTp + SC non-completers did not differ from the CBTp + SC completers on pre-therapy clinical status and neuropsychological function, whereas SC control non-completers had lower pre-therapy scores on 2 out of 12 neuropsychological measures than SC control completers (Letter-Number test number of items correctly recalled: completers = 13.22 (2.58) and non-completers = 9.30 (4.37), *F* = 9.01, df = 1,25, *p* = 0.006; Hopkins verbal learning test number of items freely recalled: completers = 22.39 (4.58) and non-completers = 17.78 (5.14), *F* = 5.62, df = 1,25, *p* = 0.026). SC control completers and non-completers did not differ on pre-therapy clinical status.

All patients received a PANSS rating ≥ 60, reported at least one positive ‘distressing’ symptom of schizophrenia and wished to receive CBTp in addition to SC. Patients were on stable doses of antipsychotic medication for at least two years and on the current antipsychotic drug for at least three months prior to taking part (86% on atypical antipsychotics). Patients in both treatment groups were recruited from the South London and Maudsley (SLAM) NHS Foundation Trust, were identified by local psychiatric consultants as suitable for CBTp and wished to receive CBTp in addition to their usual care. Patients who were referred to and accepted for CBTp by the Psychological Interventions Clinic for Outpatients with Psychosis (PICuP), SLAM NHS Foundation Trust, went into the CBTp + SC group. Ninety-five patients were referred to PICuP, of whom 31% participated in the study, 23% did not wish to take part, 21% were not considered because they were either not fluent English speakers or had received CBTp in the past, 20% were not suitable for PICuP or withdrew before assessment of suitability for PICuP, and 3% were not suitable for MRI; the reason for non-inclusion of 2% was not known (most likely they missed their appointment). Those who did not wish to take part or were excluded had a similar age on average to those who took part. Patients who matched the CBTp + SC group patients demographically and clinically as much as possible were studied as part of the SC control group over the same interval as the CBTp + SC group patients. Patients were recruited in a case-controlled manner. Although treatment allocation was not randomised, there was no discernable difference between CBTp + SC and SC control groups with regard to their suitability or desire to undergo the intervention.

CBTp was delivered by trained therapists, and followed the procedure developed by Fowler et al. (1995). CBTp, as defined by the National Institute for Health and Clinical Excellence updated guidelines (NICE, 2009), consisted of enabling the patient to: (i) establish links between their thoughts, feelings or actions with respect to the current or past symptoms, and/or functioning; and (ii) re-evaluate their perceptions, beliefs or reasoning related to the target symptoms. In addition, CBTp involved enabling the patient to monitor his/her own thoughts, feelings or behaviours with respect to the symptom or recurrence of symptoms; and/or (i) promotion of alternative ways of coping with the target symptom, (ii) the reduction of distress, and/or (iii) the improvement of functioning. Therapy sessions were conducted on a weekly or fortnightly basis, as preferred by the patient. CBTp patients received an average of 19 (S.D. 7) therapy sessions. Treatment adherence was assessed by fortnightly supervision. In addition, a number of therapy sessions (*n* = 13) were taped and rated independently by an experienced CBTp therapist for fidelity of treatment using the Cognitive Therapy Scale for Psychosis (Haddock et al., 2001). The mean rating was 40.7 (range 21–53) out of a maximum of 60, with 77% of the tapes scoring above the 50% mark (i.e. rating score > 30).

SC consisted of case management offered by the case management team for a particular geographical area. The case management included regular reviews with a dedicated care coordinator who monitored the patient's mental health needs, offered support and provided further specialist input, viz. regular reviews with a psychiatrist. The care coordinator also arranged for support from other specialists, such as a psychologist, vocational adviser, benefits adviser and occupational therapist as needed. Six-monthly care plan assessment reviews were carried out with a focus on recovery. The patients in the SC control group were not receiving any control psychological intervention.

The study procedures were approved by the ethics committee of the Institute of Psychiatry and the SLAM NHS Foundation Trust, London. All participants provided written informed consent to their participation and were compensated for their time in undergoing MRI scanning and clinical and neuropsychological assessments (participants were not paid for therapy) and travel.

### 2.2 Clinical assessments

Clinical diagnosis at baseline was confirmed by a consultant psychiatrist (DF) using the Structured Clinical Interview for DSM-IV (First et al., 2002). PANSS assessments (Kay et al., 1987) were performed on patients before and after CBTp by an experienced psychiatrist (DF). This psychiatrist played no role in patient recruitment or clinical management of the patients included in this investigation and was blind to whether or not a patient received CBTp in addition to their usual treatment. Clinical and neuropsychological assessments were carried out by doctoral-level researchers on patients at baseline and follow-up. Neuropsychological assessments in healthy participants were performed once (at baseline).

#### 2.2.1 Beck Depression Inventory-II

The Beck Depression Inventory-II (Beck, 1996) is a 21-item questionnaire assessing the main emotional, cognitive and physiological symptoms of depression (Beck et al., 1988b). The total score was used in the analysis.

#### 2.2.2 Beck Anxiety Inventory

The Beck Anxiety Inventory (Beck et al., 1988a) is a 21-item questionnaire assessing the main symptoms associated with anxiety disorder. The total score was used in the analysis.

#### 2.2.3 Rosenberg Self-esteem Scale

The Rosenberg Self-esteem Scale (Rosenberg, 1965) is a 10-item self-reported inventory. It measures overall feelings of self-worth or self-acceptance. Items are rated on a 4-point Likert Scale from ‘Strongly Agree’ to ‘Strongly Disagree’. The total score was used in the analysis.

#### 2.2.4 Beck Cognitive Insight scale

The Beck Cognitive Insight scale (Beck et al., 2004) is a 15-item self-reported inventory. The items separate into two factors, namely self-certainty and self-reflectiveness. These two factors and the composite score, which is derived from the difference between self-certainty and self-reflectiveness, were used in the analysis.

#### 2.2.5 Schedule for the Assessment of Insight — expanded

The Schedule for the Assessment of Insight — expanded (SAI-E) (Kemp and David, 1997) is a 9-item researcher administered inventory relating to the patient's awareness of illness and specific symptoms, illness attribution, awareness of illness consequences, treatment compliance, symptom re-labelling, and openness to contradiction of his/her beliefs. The total score was used in the analysis.

#### 2.2.6 Birchwood Insight Scale

The BIS (Birchwood et al., 1994) is an 8-item self-reported inventory. Items include “Some of my symptoms are made by my mind” and “The doctor is right in prescribing medication for me”. Item 4 “My stay in hospital is necessary” was disregarded, as all patients were outpatient. The total score was used in the analysis.

#### 2.2.7 Coping Orientation to Problems Experience

The Coping Orientation to Problems Experienced (COPE) inventory (Carver et al., 1989) is a 60-item self-reported coping inventory which assesses the ways in which individuals respond to stress based on the theoretical principles underlying Lazarus' model of stress (Lazarus, 1966; Lazarus and Folkman, 1984). The format used in the present study asked patients to indicate how they had been coping with the stress in their life since they were told that they had a mental health problem. The scale consists of 15 subscales, namely Positive reinterpretation and growth, Mental disengagement, Focus on and venting of emotions, Use of instrumental social support, Active coping, Denial, Religious coping, Behavioural disengagement, Restraint, Use of emotional social support, Substance use, Suppression of competing activities, Acceptance, Planning and Humour. The total and subscale scores were used in the analysis.

### 2.3 Neuropsychological assessments

#### 2.3.1 General intelligence

##### 2.3.1.1 Wechsler Abbreviated Scale of Intelligence

The Wechsler Abbreviated Scale of Intelligence (WASI, Wechsler, 1999) is designed to be a short test of general intellectual ability. The two-subtest version of the WASI consists of the Vocabulary subtest and the Matrix Reasoning subtest. The subtests were combined to estimate full-scale equivalent current IQ.

##### 2.3.1.2 National Adult Reading test

The National Adult Reading test (Nelson and Wilson, 1991) is a measure of premorbid IQ. Selection of the word list in this test is based on the existence of atypical words that do not follow the common rules of grapheme–phoneme representation and pronunciation. Predicted premorbid IQ was the variable used in the analysis.

#### 2.3.2 Executive function

##### 2.3.2.1 Behavioural Assessment of Dysexecutive Syndrome

The Behavioural Assessment of Dysexecutive Syndrome (BADS, Wilson et al., 1996) is a test of executive functioning comprising six subtests: Rule Shift Cards, Action Program Test, Temporal Judgement Test, Key Search Test, Zoo Map Test, and the Modified Six Elements. Each subtest has a range of scores from 0 to 4. Total profile score was the variable used in the analysis.

##### 2.3.2.2 Brixton Spatial Anticipation test

The Brixton Spatial Anticipation test (BSAT, Burgess and Shallice, 1997) is a test of concept formation in which the participant is presented with a sequence of 56 near-identical stimuli, each of which has 10 circles, one of which is coloured blue. The participant is instructed to point to the expected position of a coloured circle on the next page. The location of this coloured circle is determined by one of nine rules based on the previous position of the coloured circle. The number of perseverative errors was used in the analysis.

##### 2.3.2.3 Wisconsin Card Sorting test

The computerised version of the Wisconsin Card Sorting test (WCST, Heaton et al., 1993) was used. Participants are asked to match the choice-card to the upper panel of cards using a hidden rule. Successful completion of the WCST requires participants to (a) attain concept or the current rule, (b) maintain the concept for 10 consecutive trials and (c) switch the concept when the sorting rule is changed. Number of perseverative errors was used in the analysis.

#### 2.3.3 Working memory

##### 2.3.3.1 Letter Number test

In this task (Gold et al., 1997), participants are asked to recall a string of letters and numbers, placing the numbers first in ascending order, followed by the letters in alphabetical order. Total number of correct items was used in the analysis.

#### 2.3.4 Immediate verbal learning and memory

##### 2.3.4.1 Wechsler Memory Scale III — Logical Memory

In this subtest of the Wechsler Memory Scale — III (Wechsler, 1998), the participant listens to two different stories read by the examiner and is asked to retell it from memory immediately after hearing each story (immediate recall) and half an hour later (delayed recall). Immediate and delayed unit recall scaled scores were used in the analysis.

##### 2.3.4.2 Hopkins Verbal Learning test — Revised

In the free recall part of the Hopkins Verbal Learning test (Shapiro et al., 1999), the examiner reads out a list of 12 words which the participant is asked to recall. The list is read out three times and the number of words correctly recalled on each occasion is noted. Total number of items freely recalled was used in the analysis.

#### 2.3.5 Attention

##### 2.3.5.1 Continuous Performance Test — Identical Pairs (CPT-IP)

This test (Cornblatt et al., 1988) requires the participant to respond when two identical stimuli appear consecutively on the computer screen. Stimuli (four-digit numbers) are flashed on the screen at a constant rate of one stimulus per second, with a stimulus “on” time of 50 ms. Target trials are the second stimulus of a pair of identical stimuli. Discriminability index was used in the analysis.

#### 2.3.6 Verbal skills

##### 2.3.6.1 Verbal fluency test

The verbal fluency test (Milner, 1975) requires the participant to generate as many words as possible starting with a specified letter or a specified category within 60 seconds. Word generation is based on one of three letters: F, A and S, and on one of three categories: animal, fruit and vegetable. Number of correct words generated in each subtest was used in the analysis.

#### 2.3.7 Cognitive inhibition

##### 2.3.7.1 Hayling Sentence Completion Test

The Hayling Sentence Completion Test (HSCT, Burgess and Shallice, 1997) requires the participant to provide single-word completions to sentences, each of which has the last word missing. There are two parts: in part A, participants must produce sensible completions as quickly as possible; in part B, the participant must produce completions that are unrelated to the preceding sentence. The time taken to complete the two parts as well as the errors made in part B is converted into a scaled score. Hayling B scaled score was used in the analysis.

#### 2.3.8 Emotional decision-making

##### 2.3.8.1 Iowa Gambling Task

In the Iowa Gambling Task (IGT) (Bechara et al., 1994), participants choose cards from advantageous and disadvantageous decks, such that choosing from the disadvantageous decks is associated with greater immediate monetary reward compared to the advantageous decks, but an overall greater monetary loss compared to the advantageous decks. The trials are presented in five blocks. Overall learning (score on Block 5 minus Block 1) was used in the analysis.

### 2.4 Statistical analysis

#### 2.4.1 Comparison of patient group characteristics at baseline

Due to the large number of neuropsychological measures (*n* = 12), individual test scores were combined within each domain to provide composite domain scores at baseline and follow-up (see Section 2.3 neuropsychological assessments for tests included in each domain) based on the approach followed by Kern et al. (2008). The patient groups were compared on gender using Chi-squared test, and baseline clinical and neuropsychological domain variables using analysis of variance or Mann–Whitney U-tests for variables with significant heterogeneity of variance (number of years in education, duration of illness, Birchwood Insight Scale total score, working memory, verbal skills and emotional decision-making domain scores).

#### 2.4.2 Change in clinical status and neuropsychological ability from baseline to follow-up

Absolute change from baseline to follow-up was computed for each clinical and neuropsychological domain variable (calculated so that a negative change score indicated improvement in clinical status or neuropsychological function at follow-up). For all clinical measures (except BCI composite index subscale), a higher score on the scale indicates greater severity. Therefore, change on these clinical measures was calculated as follow-up score minus baseline score (except for BCI composite index subscale, where change was calculated as baseline score minus follow-up score). For all neuropsychological domain scores, a higher score indicates better performance. Therefore, change on neuropsychological domain scores was calculated as baseline score minus follow-up score. Analyses of co-variance were carried out in order to compare the change in clinical and neuropsychological domain scores in the two patient groups with baseline clinical or neuropsychological domain score as the covariate. For variables with significant heterogeneity of variance in the change scores between patient groups (verbal skills), Mann–Whitney U tests were performed.

#### 2.4.3 Pre-therapy clinical predictors of symptom change

Analysis of variance with gender as the independent variable and residual change in PANSS total, positive, negative and general psychopathology symptom ratings as the dependent variable was performed in the CBTp + SC group. For the purpose of this analysis, symptom change was estimated as the residual change in PANSS symptoms (change in symptom scores covarying for baseline symptom scores) (Siegle et al., 2006). Although other variables were also found to have changed significantly at follow-up (Results, Section 3.2 ‘Change in clinical status and neuropsychological ability following CBTp’), given the modest sample sizes we limited our analyses to predictors of psychotic symptom change. Pearson correlations, or Spearman correlations for variables with significant kurtosis or skewness (antipsychotic medication level and Birchwood Insight total score), were performed between pre-therapy clinical variables and symptom change scores in the CBTp + SC and SC control groups separately. In order to determine whether the pre-therapy clinical variables found to significantly predict CBTp responsiveness had a differential effect on outcome in the CBTp + SC relative to SC control group, correlations were repeated in the SC control group for those correlations found to be significant in the CBTp + SC group. The correlation coefficients from the two groups were compared using Fisher's *z* transformation (Howell, 2002).

Finally, Pearson correlations were performed between number of CBTp therapy sessions and symptom change scores in order to examine whether this factor was related to treatment outcome.

As the aforementioned correlational analyses showed that pre-therapy COPE total, BCI self-reflectiveness and BCI composite index scores correlated with symptom change following CBTp (Results, Section 3.3), multiple regression analyses were performed to determine which pre-therapy clinical variable contributed more strongly to the variance in the criterion variable (symptom change) in the CBTp + SC group. Predictor variables (pre-therapy COPE total, BCI self-reflectiveness and BCI composite index scores) were entered in a stepwise manner (to estimate the effect of individual predictors on the variability of the criterion variable) and using a standard regression (all predictors were entered simultaneously and results were given for only one step to explain the combined effect of predictors on the variability of the criterion variable).

#### 2.4.4 Pre-therapy neuropsychological correlates of symptom change

Pearson correlations were performed between pre-therapy neuropsychological domain scores and symptom change scores following CBTp in CBTp + SC patients.

All analyses were performed in SPSS (version 15). Alpha level for testing significance of effects was maintained at *p* ≤ 0.05.

## 3 Results

### 3.1 Characteristics of patient groups at baseline

**Fig. 1:**
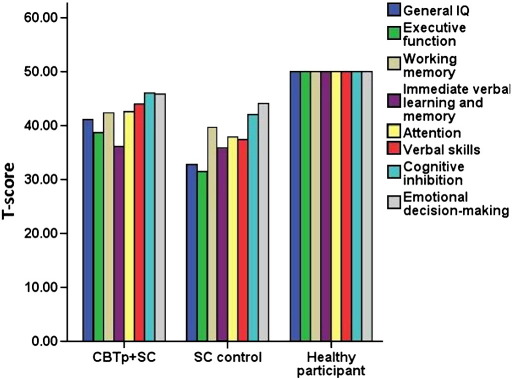
Pre-therapy standardized T-scores of neuropsychological domains in CBTp + SC (*n* = 25), SC control (*n* = 18) and healthy participant groups (*n* ranging from 19 to 25 in different domains).

The CBTp + SC group had lower BCI self-certainty, greater BCI composite index, and greater SAI total scores than the SC control group at baseline (see Table 1). The CBTp + SC group had higher general IQ and verbal skills and tended to have better attention than the SC control group at baseline (see Table 1 and Fig. 1).

### 3.2 Change in clinical status and neuropsychological ability following CBTp

The CBTp + SC group showed greater improvement from baseline to follow-up than the SC control group on total, positive, negative and general psychopathology symptom ratings, as well as self-reported depression and self-esteem, with a trend towards improvement in BCI self-certainty (lower self-certainty) (see Table 2).

There were no significant differences in outcome between the groups on anxiety, clinical insight and BCI composite index, coping skills, or any of the neuropsychological domains (see Table 2).

### 3.3 Pre-therapy clinical and neuropsychological predictors of CBTp responsiveness

There was no effect of gender on symptom change in the CBTp + SC group (total symptoms change, *F* = 0.241, df = 1,11, *p* = 0.632; positive symptoms change, *F* < 0.001, df = 1,11, *p* = 0.990; negative symptoms change, *F* = 0.151, df = 1,11, *p* = 0.700; general psychopathology change, *F* = 1.593, df = 1,11, *p* = 0.233).

Greater BCI self-reflectiveness scores at baseline correlated with improvement in total symptom ratings in the CBTp + SC group (*r* = 0.409, *p* = 0.042), but not the SC control group (*r* = − 0.180, *p* = 0.475) (see Table 3). These correlation coefficients differed between groups (*z* = 1.845, *p* = 0.033). Greater BCI self-reflectiveness at baseline also correlated with improvement in negative symptoms in the CBTp + SC group (*r* = 0.419, *p* = 0.037), but not the SC control group (*r* = − 0.054, *p* = 0.831). These correlation coefficients differed between groups at a trend level (z = 1.487, *p* = 0.068).

Greater BCI composite scores at baseline correlated with improvement in total symptoms in the CBTp + SC group (*r* = 0.419, *p* = 0.037), but not in the SC control group (*r* = − 0.227, *p* = 0.364). These correlation coefficients differed between groups (*z* = 2.022, *p* = 0.020). Greater BCI composite scores at baseline also correlated with improvement in general psychopathology symptoms in the CBTp + SC group (*r* = 0.426, *p* = 0.034), but not in the SC control group (*r* = − 0.155, *p* = 0.540). Again, these correlation coefficients differed between groups (*z* = 1.822, *p* = 0.030).

Greater coping ability at baseline correlated with improvement in total symptoms in the CBTp + SC group (*r* = 0.496, *p* = 0.022), but not in the SC control group (*r* = 0.043, *p* = 0.874).^2^ These correlation coefficients differed between groups at a trend level (z = 1.382, *p* = 0.085). Greater coping ability at baseline also correlated with improvement in negative symptoms in the CBTp + SC group (*r* = 0.450, *p* = 0.041), but not in the SC control group (*r* = − 0.056, *p* = 0.838). These correlation coefficients differed between groups at a trend level (z = 1.4842, *p* = 0.069). Lastly, greater coping ability at baseline correlated with improvement in general psychopathology symptoms in the CBTp + SC group (*r* = 0.424, *p* = 0.055), but not in the SC control group (*r* = 0.168, *p* = 0.533). These correlation coefficients did not differ between groups (z = 0.788, *p* = 0.215).

In the CBTp + SC group, there were no significant correlations between symptom change from baseline to follow-up and illness duration, age of onset, level of antipsychotic medication, self-reported depression and anxiety, clinical insight, self-esteem, BCI self-certainty, number of therapy sessions and any of the neuropsychological domains at baseline (see Table 3).

A stepwise multiple regression (stepwise model: multiple *R* = 0.496, *F* = 6.215, *p* = 0.022) of pre-therapy COPE total, BCI self-reflectiveness and BCI composite index scores on total symptom change scores in the CBTp + SC group revealed that COPE total was a significant predictor and explained 20.7% of the variance in total symptom change. Together (standard regression model: multiple *R* = 0.577, *F* = 2.835, *p* = 0.069), the three pre-therapy clinical predictors together explained 21.6% of the variance in total symptom change.

A stepwise multiple regression (stepwise model: multiple *R* = 0.450, *F* = 4.828, *p* = 0.041) of pre-therapy COPE total and BCI self-reflectiveness scores on negative symptom change scores in the CBTp + SC group revealed that COPE total was a significant predictor and explained 16.1% of the variance in negative symptom change. Together (standard regression model: multiple *R* = 0.521, *F* = 3.359, *p* = 0.058), the two pre-therapy clinical predictors explained 19.1% of the variance in negative symptom change.

The standard, but not the stepwise, multiple regression of pre-therapy COPE total and BCI composite scores on general psychopathology symptom change in the CBTp + SC group was significant (multiple *R* = 0.532, *F* = 3.550, *p* = 0.050). Pre-therapy COPE total and BCI composite index scores together explained 20.3% of the variance in general psychopathology severity change.

## 4 Discussion

The present study investigated (a) the pre-therapy clinical and neuropsychological predictors of CBTp and (b) the change in clinical status and neuropsychological function following CBTp. The findings from these investigations will be discussed in turn.

### 4.1 Clinical and neuropsychological predictors of CBTp responsiveness

Three pre-therapy clinical variables emerged as significant predictors of total symptom improvement following CBTp, namely coping, and two out of the three Cognitive Insight Scale (Beck et al., 2004) scores, namely self-reflectiveness and the composite index score (being able to self-reflect while refraining from overconfidence in one's judgements). Pre-therapy cognitive insight and coping predicted symptom improvement more strongly in the CBTp + SC than the SC control group, suggesting that they are specific predictors of CBTp responsiveness rather than of good outcome in general, although the size of the difference in the coping-symptom change relation between the CBTp and SC control groups was weaker than the cognitive insight-symptom change relation. Therefore, coping and cognitive insight (participants' intrinsic ability to reappraise misinterpretations), rather than clinical insight, duration of illness, gender or neuropsychological function, appeared to be the important factors in predicting symptom improvement following CBTp. However, these relationships were significant for negative and general, but not positive, symptom improvement.

Pre-therapy coping was found to be the strongest of the three significant predictors of change. Several pre-therapy coping strategies were associated with CBTp responsiveness (e.g. active coping, planning and use of emotional social support). The pattern of our findings is consistent with the view that patients with schizophrenia who have a range of coping strategies and are flexible about their coping responses are more likely to experience psychological well-being (Phillips et al., 2009). Active coping is the process of taking active steps to try to remove or circumvent the stressor or ameliorate its effects (Carver et al., 1989). Active coping includes initiating direct action, increasing one's efforts and trying to execute a coping attempt in stepwise fashion (Carver et al., 1989). Planning is thinking about how to cope with a stressor. Planning involves coming up with action strategies, thinking about what steps to take and how best to handle the problem. Being able to plan a coping response and then actively carry out the response may moderate CBTp's aim to alleviate general distress (i.e. reduce general symptoms) and increase functioning (i.e. reduce negative symptoms), although it does not appear to be related to outcome for positive symptoms.

#### 4.1.1 Insight and CBTp responsiveness

The ability to reflect on one's anomalous experiences (self-reflectiveness) before therapy predicted improvement in negative symptoms in the CBTp + SC, but not the SC control, group. Being able to self-reflect while refraining from overconfidence in one's judgements (composite index) before therapy predicted improvement in general psychopathology in the CBTp + SC group, but not the SC control group. These findings support and extend those of Perivoliotis et al. (2010) and Garety et al. (1997) by demonstrating that cognitive insight or flexibility may be related to improvements in negative and general symptoms. Having some ability to re-evaluate perceptions, beliefs and reasoning beforehand may therefore benefit CBTp responsiveness in a number of different areas. Greater self-reflectiveness is associated with lower severity of positive and negative symptoms (Bora et al., 2007). In the present study, overconfidence about one's interpretations per se did not predict symptom improvement. This lack of a relationship may be because the level of delusional beliefs in our group of patients before therapy may have been at a moderate level, and overconfidence about judgements, which may be equivalent to the jumping-to-conclusions (JTC) reasoning style, is thought to underlie delusional beliefs (Garety and Freeman, 1999). In one study (Warman and Martin, 2006), patients with schizophrenia or schizoaffective disorder who had active delusions tended to endorse their beliefs and judgements more readily than healthy participants who were delusion-prone and healthy participants with no delusional proneness, i.e. the patients with active delusions expressed more confidence in their judgements about the emotional salience of personally relevant statements. Brabban et al. (2009) found that a lower level of conviction about delusions predicted a greater likelihood of improvement in general psychopathology only in patients with delusions. The present study and an earlier study (Perivoliotis et al., 2010) suggest that the composite index is different conceptually from the individual subscales and shed light on the nature of the Cognitive Insight scale. The results suggest that refraining from overconfidence in one's judgements on its own does not predict symptom improvement, but does in the context of being able to self-reflect. The composite index takes into account the fact that the two subscales are not mutually exclusive (Beck et al., 2004; Warman et al., 2007).

In the multiple regression analyses of pre-therapy predictors of CBTp responsiveness, pre-therapy cognitive insight did not add significantly to the variance of the model explained by pre-therapy coping. This may have been because of the similar processes involved in coping and cognitive insight, as suggested by the overlapping variances of these variables. Clinical insight and coping have already been shown to work congruently in alleviating symptoms in patients with psychosis (Cooke et al., 2007; Lysaker et al., 2005; Phillips et al., 2009). Those who are able to successfully ‘integrate’ their illness with their view of themselves are more likely to develop successful coping strategies than those who ‘seal over’ and perceive their illness as negative and interrupting the progress of their lives (Phillips et al., 2009). Cooke et al. (2007) observed a range of associations between clinical insight and coping. Specifically, better use of instrumental support and planning were associated with better illness insight in schizophrenia patients, and lesser mental disengagement and better ability to suppress competing activities with better awareness of problem. Unlike previous studies (Naeem et al., 2008; Emmerson et al., 2009), we did not find pre-therapy clinical insight to predict symptom improvement following CBTp. However, the Insight trial (Naeem et al., 2008) was specifically targeting insight as a treatment goal, and Emmerson et al. (2009) were targeting independent living skills, unlike the therapy delivered in this study, which was more similar to that implemented by Garety et al. (1997), who also did not find that clinical insight was a predictor of change.

In the present study, pre-therapy neuropsychological functioning did not predict CBTp responsiveness, which is mainly consistent with previous studies (Garety et al., 1997; Penades et al., 2010). Although Penades et al. (2010) reported verbal memory to predict improvement in positive symptoms, their sample comprised patients with a slightly longer duration of illness [mean (S.D.) = 14.9 (1.2)] than our sample [mean (S.D.) = 11.73 (7.89)]. Penades et al. (2010) also used a categorical approach to define whether patients were ‘improvers’ or ‘non-improvers’, while the present study's comparison consisted of outcome in people receiving CBTp + SC versus SC. We earlier reported (Premkumar et al., 2009) that CBTp + SC responders had larger posterior hippocampal volume than CBTp + SC non-responders, which given that greater hippocampal volume is related to better learning and memory in schizophrenia patients (Antonova et al., 2004; Nestor et al., 2007; Rametti et al., 2007), is consistent with the Penades et al. study findings. Specific measures of cognitive control and self-versus-other discrimination may also be more relevant to CBTp response (Kumari et al., 2009; Kumari et al., 2010). Future studies may also examine whether pre-therapy neuropsychological measures related to cognitive insight, such as the meta-cognition task (Lysaker et al., 2009), are related to symptom improvement following CBTp. We also did not observe the effect of gender or duration of illness on symptom improvement that has been previously reported (Tarrier et al., 1998; Brabban et al., 2009). It is possible that gender effects may be specific to certain CBTp formats, as women responded better to brief CBTp (6 sessions over 3 months) than men (Brabban et al., 2009), and duration of illness may predict CBTp when therapy is combined with coping skills enhancement (Tarrier et al., 1998).

### 4.2 Changes in clinical status following CBTp

The levels of depression and self-esteem and, at a near significant level, self-certainty improved to a greater extent in the CBTp + SC than SC control group, in addition to PANSS symptoms. Our findings support a number of studies demonstrating significant improvements in depression (Garety et al., 2008; Peters et al., 2010) and increased self-esteem (Gumley et al., 2006). Although self-certainty did not predict CBTp responsiveness, possibly because such an association may be observed in patients with a greater level of delusions (Brabban et al., 2009), it somewhat improved following CBTp in the present study. CBTp may help patients to refrain from overconfidence in their beliefs and inferences of anomalous experiences regardless of the presence of delusional beliefs.

### 4.3 Limitations and future research

The moderate sample sizes of the CBTp + SC and SC control groups may not have allowed sufficient power to demonstrate significant group differences in the pre-therapy coping-symptom improvement relationships. This study, like most studies on coping strategies in psychosis (Phillips et al., 2009), used a relatively small number of outpatients who mostly had a paranoid schizophrenia diagnosis. This sample represents a relatively stable population of patients who have greater access to social and community-based systems than would their inpatient counterparts, and may use different coping strategies to inpatients (Phillips et al., 2009). Further research is needed to test this possibility. In addition to patients with schizophrenia, CBTp is found to be effective in patients in the early initial prodromal state of the pre-psychotic phase (Morrison et al., 2004; Bechdolf et al., 2005). Future research should also analyse the role of cognitive insight and coping in the efficacy of CBTp in pre-psychotic phase.

The CBTp + SC group had greater pre-therapy cognitive insight, general IQ and verbal skills than the SC control group at baseline, which may have contributed to their better outcome, and confounded the pre-therapy cognitive insight-symptom change correlations. It is unlikely that the CBTp group would have improved to the same extent without CBTp over the course of this study, but it is possible that the SC control group would show less improvement with CBTp when offered as a result of lower pre-therapy level of insight. A greater number of drop-outs in the SC control, than CBTp + SC, group may also have led to a bias in some of the findings.

### 4.4 Conclusions

Coping styles and cognitive insight before therapy play a role in determining CBTp efficacy in patients with schizophrenia, specifically for negative and general symptoms. Being able to reflect on one's psychotic experiences and reappraise misinterpretations while refraining from being overconfident about one's interpretations, as well as having coping strategies, especially planning and active coping, are related to better CBTp responsiveness.

## Conflict of interest

The authors declare no conflict of interest.

## Figures and Tables

**Table 1:** Clinical and neuropsychological characteristics at baseline in CBTp + SC and SC control groups.

	CBTp + SC (*n* = 25)			SC control (*n* = 18)			*F* or *Z*^†^	Statistic	df	*p*
	*N*	Mean	S.D.	*N*	Mean	S.D.				
*Characteristic*										
Gender (number of males/females)	25	17/8		18	15/3					
Age	25	36.08	8.02	18	39.72	10.49	*F*	1.688	1,41	0.204
Years in education	25	14.04	2.91	18	13.28	1.93	*Z*	0.696	1,41	0.487
Duration of illness	25	11.73	7.89	18	14.50	12.10	*Z*	0.444	1,41	0.657
Age of onset	25	24.36	8.01	18	25.22	8.24	*F*	0.188	1,41	0.733
Medication dosage— CPZ equivalent	25	526.15	385.78	18	459.65	336.47	*F*	0.345	1,41	0.560
PANSS symptoms at baseline										
Positive	25	17.96	4.74	18	18.33	3.45	*F*	0.081	1,41	0.778
Negative	25	17.84	4.36	18	19.05	3.89	*F*	0.888	1,41	0.352
General psychopathology	25	32.72	7.21	18	35.50	4.19	*F*	2.146	1,41	0.151
Total	25	68.52	13.53	18	72.89	9.01	*F*	1.417	1,41	0.241
BDI	24	15.75	8.29	18	16.44	10.18	*F*	0.059	1,40	0.809
BAI	25	14.16	10.81	18	20.06	12.20	*F*	2.795	1,41	0.102
Rosenberg self-esteem	25	23.88	6.24	17	23.18	5.68	*F*	0.138	1,40	0.712
BCI self-reflectiveness	25	16.28	5.81	18	13.44	5.40	*F*	2.643	1,41	0.112
BCI self-certainty	25	5.84	3.58	18	8.44	3.60	*F*	**5.514**	**1,41**	**0.024**
BCI composite	25	10.44	7.17	18	5.00	7.04	*F*	**6.122**	**1,41**	**0.018**
SAI total	24	13.18	5.88	17	10.61	5.24	*F*	**4.767**	**1,39**	**0.035**
Birchwood insight total	25	9.68	2.14	18	5.17	2.12	*Z*	1.796	1,41	0.072
COPE total	21	73.86	28.15	16	82.87	29.75	*F*	0.888	1,35	0.353

*Neuropsychological domain*										
General intelligence	25	41.13	12.31	18	32.75	14.88	*F*	**4.074**	**1,41**	**0.050**
Executive function	25	38.63	19.60	18	31.45	10.86	*F*	1.968	1,41	0.168
Working memory	25	42.21	12.55	18	39.49	8.19	*Z*	0.496	1,41	0.620
Immediate verbal learning and memory	25	36.12	9.18	18	35.98	8.78	*F*	0.003	1,41	0.959
Attention	25	42.54	8.48	18	37.91	6.75	*F*	3.681	1,41	0.062
Verbal skills	25	44.01	8.64	18	37.40	5.75	*Z*	**2.511**	**1,41**	**0.012**
Cognitive inhibition	25	46.03	11.99	18	42.04	13.95	*F*	1.014	1,41	0.320
Emotional decision-making	25	45.82	13.08	18	44.11	8.73	*Z*	< 0.001	1,41	1.000

Values in bold: significant difference between patient groups at *p* ≤ 0.05; ^†^Mann–Whitney *U* test (*Z* statistic) performed for variables with significant variance heterogeneity between groups.

**Table 2:** Change in clinical and neuropsychological status from baseline to follow-up in CBTp + SC and SC control groups controlling for clinical status at baseline.

	CBTp + SC			SC control			*F* or *Z*^†^	Statistic	*p*
	*N*	Mean	S.D.	*N*	Mean	S.D.			
*Clinical variable*									
PANSS symptoms									
Positive	25	− 3.04	4.05	18	− 0.56	3.79	*F*	**6.246**	**0.017**
Negative	25	− 2.36	3.81	18	0.72	4.67	*F*	**9.551**	**0.004**
General psychopathology	25	− 4.68	7.42	18	− 0.83	6.70	*F*	**6.772**	**0.013**
Total symptoms	25	− 10.08	12.93	18	− 0.67	12.19	*F*	**8.664**	**0.005**
BDI	23	− 5.57	10.17	17	0.76	7.50	*F*	**4.775**	**0.035**
BAI	23	− 4.26	10.71	17	− 3.18	12.61	*F*	2.864	0.099
Rosenberg self-esteem	25	− 1.04	6.15	17	2.25	3.84	*F*	**4.069**	**0.051**
BCI self-reflectiveness	25	− 1.48	4.69	16	<−0.001	4.26	*F*	0.045	0.833
BCI self-certainty	25	− 1.52	3.87	16	− 0.37	3.67	*F*	3.791	0.059
BCI composite	25	0.04	3.55	17	0.37	6.16	*F*	0.398	0.532
SAI total^‡^	14	0.89	5.46	9	2.63	3.61	*F*	0.710	0.409
Birchwood insight total	25	− 0.27	1.80	16	− 0.27	1.87	*F*	0.244	0.624
COPE total	21	− 2.00	27.28	13	− 0.77	20.55	*F*	0.531	0.472

*Neuropsychological domain*									
Executive function	18	− 2.40	16.57	13	− 7.83	12.98	*F*	0.494	0.488
Working memory	24	− 2.82	10.10	17	− 1.81	7.46	*F*	0.681	0.414
Immediate verbal learning and memory	25	− 5.55	7.75	17	− 3.23	7.50	*F*	1.160	0.288
Attention	24	− 1.30	7.55	17	− 2.68	7.94	*F*	0.033	0.856
Verbal skills	24	− 1.28	5.13	17	− 1.19	3.25	*Z*	0.132	0.895
Cognitive inhibition	24	− 2.22	11.94	17	− 1.39	20.66	*F*	0.535	0.469
Emotional decision-making	23	− 0.35	16.42	17	1.82	11.83	*F*	2.288	0.139

Values in bold: significant difference between patient groups at *p* ≤ 0.05; negative mean score indicates improvement in clinical or neuropsychological status; † Mann–Whitney *U* test (*Z* statistic) performed for variables with significant variance heterogeneity between groups; ‡ SAI total score at follow-up was available only for patients who had a score ≥ 4 on a PANSS positive item.

**Table 3:** Correlation between baseline clinical status/neuropsychological function and symptom change in CBTp + SC patients.

	*N*	PANSS total	PANSS positive	PANSS negative	PANSS general psychopathology
*Baseline clinical variable*					
Duration of illness	25	− 0.066 (0.753)	− 0.092 (0.661)	− 0.096 (0.647)	− 0.047 (0.823)
Age of onset	25	0.380 (0.061)	0.305 (0.139)	0.247 (0.235)	0.379 (0.062)
† Medication dosage (CPZ equivalent)	25	0.019 (0.929)	− 0.152 (0.469)	0.329 (0.109)	0.075 (0.722)
BDI	24	0.179 (0.402)	0.087 (0.685)	0.338 (0.107)	0.122 (0.569)
BAI	25	0.077 (0.716)	0.062 (0.768)	0.026 (0.902)	− 0.007 (0.975)
Rosenberg Self-Esteem	25	0.176 (0.399)	0.068 (0.747)	0.371 (0.068)	0.113 (0.592)
BCI self-reflectiveness	25	**0.409 (0.042)**	0.210 (0.313)	**0.419 (0.037)**	0.376 (0.064)
BCI self-certainty	25	− 0.175 (0.401)	− 0.240 (0.247)	− 0.009 (0.966)	− 0.244 (0.240)
BCI composite	25	**0.419 (0.037)**	0.290 (0.159)	0.344 (0.092)	**0.426 (0.034)**
SAI total	24	0.094 (0.663)	0.316 (0.132)	− 0.080 (0.711)	0.063 (0.771)
† Birchwood insight total	25	− 0.176 (0.401)	0.263 (0.204)	− 0.134 (0.524)	− 0.150 (0.475)
COPE total	21	**0.496 (0.022)**	0.378 (0.091)	**0.450 (0.041)**	**0.424 (0.055)**
Number of therapy sessions	22	− 0.116 (0.608)	− 0.137 (0.543)	0.083 (0.715)	− 0.200 (0.373)

*Neuropsychological domain*					
General intelligence	25	− 0.385 (0.057)	− 0.318 (0.121)	− 0.326 (0.112)	− 0.333 (0.104)
Executive function	25	− 0.006 (0.976)	0.017 (0.934)	− 0.057 (0.788)	0.015 (0.945)
Working memory	25	− 0.176 (0.401)	− 0.248 (0.233)	− 0.104 (0.621)	− 0.171 (0.413)
Immediate verbal learning and memory	25	− 0.120 (0.569)	− 0.125 (0.550)	0.048 (0.820)	− 0.170 (0.415)
Attention	25	− 0.290 (0.159)	− 0.339 (0.097)	− 0.185 (0.375)	− 0.231 (0.266)
Verbal skills	25	− 0.055 (0.793)	0.007 (0.973)	− 0.081 (0.700)	− 0.012 (0.954)
Cognitive inhibition	25	− 0.213 (0.306)	− 0.082 (0.698)	− 0.189 (0.366)	− 0.289 (0.161)
Emotional decision-making	25	0.168 (0.422)	0.179 (0.393)	0.185 (0.377)	0.150 (0.473)

Values in bold: significant correlation at *p* ≤ 0.05; † Spearman's rho correlation performed for this variable.

## Appendix A Supplementary data

Summary of studies examining the clinical and neuropsychological predictors of CBT outcome.

## Footnotes

2 The following COPE subscales correlated positively with improvement in total symptoms in the CBTp + SC group: active coping (Spearman's rho = 0.657, *p* = 0.001), suppression of competing activities (rho = 0.485, *p* = 0.026) and acceptance (rho = 0.440, *p* = 0.046). The following COPE subscales correlated positively with improvement in negative symptoms: planning (rho = 0.596, *p* = 0.004), active coping (rho = 0.473, *p* = 0.030) and use of emotional social support (rho = 0.465, *p* = 0.034). The following COPE subscales correlated positively with improvement in general psychopathology symptoms: planning (rho = 0.671, *p* = 0.001), active coping (rho = 0.498, *p* = 0.022) and suppression of competing activities (rho = 0.489, *p* = 0.024). Behavioural disengagement correlated negatively with improvement in general psychopathology symptoms (rho = − 0.430, *p* = 0.052).
