# Supplementary material for: Coping styles predict responsiveness to cognitive behaviour therapy in psychosis
Source: Psychiatry Res. 2011 May 30;187(3-2):354–62. doi: 10.1016/j.psychres.2010.12.029 (PMC3081067; doi:10.1016/j.psychres.2010.12.029)
Supplement: Supplementary file 1 — Summary of studies examining the clinical and neuropsychological predictors of CBT outcome. [file mmc1.doc]

Supplementary material. Summary of studies examining the clinical and neuropsychological predictors of CBT outcome.

| **Study reference** | **Intervention (n = number of participants)** | **Study design** | **Number of CBT sessions and duration of CBT** | **Participants** | **Baseline predictor measure** | **Clinical outcome measure** | **Result** |
| --- | --- | --- | --- | --- | --- | --- | --- |
| **Clinical predictors** | | | | | | | |
| Tarrier et al. (1993) | 1. Coping strategy enhancement (n=15)  2. Problem-solving (n=12)  3. Waiting-list control (n=14) | RCT | Duration of intervention was six weeks | Outpatients with schizophrenia with persistent hallucinations or delusions for at least 6 months (mean age=42.7 years) | Total symptom severity score as measured by the BPRS of each individual symptom and number of symptoms score as measured by the present state examination | Change in BPRS total symptom severity score and number of symptoms score | Higher total symptom severity correlated with greater change in severity. This effect was stronger in the coping strategy enhancement than problem solving group. Higher total symptom severity and number of symptoms scores correlated with greater change in number of symptoms, though the effect was equivocal between patient groups. |

Supplementary material continued

| **Study reference** | **Intervention (n = number of participants)** | **Study design** | **Number of CBT sessions and duration of CBT** | **Participants** | **Baseline predictor measure** | **Clinical outcome measure** | **Result** |
| --- | --- | --- | --- | --- | --- | --- | --- |
| Garety et al. (1997) | 1. CBTp (n=28)  2. SC (n=32) | RCT | 19 one-hour weekly sessions over nine months | Inpatients and outpatients with medication-resistant schizophrenia with at least one positive psychotic symptom which was persistent and distressing (mean age=40 years) | Symptoms: BPRS, BDI, BAI, BHS, Delusions only, Self-esteem, delusional conviction, delusional preoccupation, delusional distress, social functioning scale.  Insight: Amador Insight Scale, MADS | Pre- to post-treatment change in BPRS score | In the CBT group, better insight into delusions (acknowledging that another view of the delusion may be possible) and more number of admissions in five years were associated with better clinical outcome.  In the SC group, higher distress levels on the BHS and poorer social functioning were associated with better clinical outcome. |

Supplementary material continued

| **Study reference** | **Intervention (n = number of participants)** | **Study design** | **Number of CBT sessions and duration of CBT** | **Participants** | **Baseline predictor measure** | **Clinical outcome measure** | **Result** |
| --- | --- | --- | --- | --- | --- | --- | --- |
| Tarrier et al. (1998) | 1. Intensive CBTp (coping strategy enhancement) + routine care (n=24)  2. Supportive counselling + routine care (n=21)  3. Routine care alone (n=27) | RCT | Twice weekly one-hour sessions over 10 weeks. | Patients with schizophrenia or schizoaffective disorder (inpatient or outpatient not specified) with persistent hallucinations or delusions for at least 6 months and stabilized for 1 month (mean age 38.6 years) | Total symptom severity score as measured by the BPRS of each individual symptom and number of symptoms score as measured by the present state examination | Good (50% or more improvement in psychotic symptoms) or poor outcome | CBT group status, a shorter duration of illness and less severity of psychotic symptoms at baseline predicted a greater likelihood of good outcome. |

Supplementary material continued

| **Study reference** | **Intervention (n = number of participants)** | **Study design** | **Number of CBT sessions and duration of CBT** | **Participants** | **Baseline predictor measure** | **Clinical outcome measure** | **Result** |
| --- | --- | --- | --- | --- | --- | --- | --- |
| Naeem et al. (2008, Insight trial) | 1. CBTp (n=225)  2. SC (n=128) | RCT | Six sessions over 3 months. The main carer was also offered 3 sessions along with carer-oriented information | Outpatients with schizophrenia with medication-resistant schizophrenia (mean age not reported) | Age, gender, illness duration, scores on CPRS, HoNOS, delusions rating scale, auditory hallucinations rating scale, brief anxiety scale, MADRS, insight scale, dosage of antipsychotics and positive and negative symptoms scale | Good (25% or more improvement in CPRS total score) versus poor outcome | CBT group status, higher insight, anxiety and scores on CPRS global rating system predicted and low scores on the delusion rating scale good outcome. |
| Naeem et al. (2008, London and Newcastle trial) | 1. CBTp (n=46)  2. Befriending (n=44) | RCT | Not reported | Inpatients and outpatients with treatment-resistant schizophrenia with persistent symptoms for 6 months causing distress (mean age not reported) | Age, gender, chronicity, overall psychopathology, depression, anxiety, negative and positive symptoms, side effects and medication received | Good (25% or more improvement in CPRS total score) versus poor outcome | There were no significant predictors of good outcome. |

Supplementary material continued

| **Study reference** | **Intervention (n = number of participants)** | **Study design** | **Number of CBT sessions and duration of CBT** | **Participants** | **Baseline predictor measure** | **Clinical outcome measure** | **Result** |
| --- | --- | --- | --- | --- | --- | --- | --- |
| Brabban et al. (2009) | 1. Brief CBTp (n=226)  2. TAU (n=128) | RCT | 6 hourly sessions over 2-3 months. Carers also received 3 sessions of education about CBT | Outpatients with schizophrenia | Gender, whether a schizophrenia diagnosis was made in the last 2 years, affective blunting, alogia and insight | 25% or greater improvement on CPRS and the Insight scale | In the CBT group, being female predicted a greater likelihood of overall symptom reduction and improvement in insight. In patients with delusions (n=211), being female and having a lower level of conviction about delusions predicted a greater likelihood of overall symptom reduction.  In the TAU group, having a schizophrenia diagnosis <2 years predicted a greater likelihood of overall symptom reduction. |

Supplementary material continued

| **Study reference** | **Intervention (n = number of participants)** | **Study design** | **Number of CBT sessions and duration of CBT** | **Participants** | **Baseline predictor measure** | **Clinical outcome measure** | **Result** |
| --- | --- | --- | --- | --- | --- | --- | --- |
| Emmerson et al. (2009) | 1. CBTp + Social skills training (n=31)  2. SC (n=31) | RCT | 24 weekly two-hour group psychotherapy sessions | Outpatients with schizophrenia or schizoaffective disorder (mean age=53 years) | Total score on the BIS | Score at follow-up on the ILSS | Greater insight at baseline predicted better functional outcome on the ILSS at 12-month follow-up after the end of treatment. |
| Perivoliotis et al. (2010) | 1. CBTp (n=78) | Cohort study | Weekly or fortnightly sessions over an average of 8 months | Outpatients with psychosis (73% with schizophrenia or schizoaffective disorder) | Self-reflectiveness, self-certainty and composite index scores on the BCI scale and severity of delusions and auditory hallucinations on PSYRATS | Residual pre- to post-treatment change in BCI and PSYRATS delusions and auditory hallucinations | Greater cognitive insight (self-reflectiveness and composite index scores) at baseline correlated with greater reductions in severity of delusions at the end of treatment. |

Supplementary material continued

| **Study reference** | **Intervention (n = number of participants)** | **Study design** | **Number of CBT sessions and duration of CBT** | **Participants** | **Baseline predictor measure** | **Clinical outcome measure** | **Result** |
| --- | --- | --- | --- | --- | --- | --- | --- |
| **Neuropsychological predictors** | | | | | | | |
| Garety et al. (1997) | 1. CBTp (n = 28)  2. SC (n = 32) | RCT | 19 one-hour weekly sessions over nine months. | Inpatients and outpatients with medication-resistant schizophrenia with at least one positive psychotic symptom which was persistent and distressing (mean age=40 years). | Cognitive Estimates test, verbal fluency – categories and letters, probabilistic reasoning, NART IQ, Quick Test | Pre- to post-treatment change in BPRS score | None was related to outcome in the CBT group, apart from, surprisingly, more errors on the Cognitive Estimates test, which were associated with better clinical outcome. |

Supplementary material continued

| **Study reference** | **Intervention (n = number of participants)** | **Study design** | **Number of CBT sessions and duration of CBT** | **Participants** | **Baseline predictor measure** | **Clinical outcome measure** | **Result** |
| --- | --- | --- | --- | --- | --- | --- | --- |
| Granholm et al. (2008) | 1. CBTp + Social skills training (n=33)  2. SC (n=32) | RCT | 24 weekly two-hour group psychotherapy sessions | Outpatients with schizophrenia or schizoaffective disorder (mean age=53.3 years). | Neuropsycholog-ical measures of speed of processing, executive functioning, attention and vigilance, and verbal learning and memory | Functional outcome as measured by the ILSS, Skill acquisition and Comprehensive Module tests | Overall neuropsychological impairment, attention and vigilance and speed of processing predicted functional outcome across both patient groups, but the size of these relationships did not differ between groups.  In the CBT group, global neuropsychological function was significantly positively associated with percentage of homework assignments completed and level of participation in group discussion. |

Supplementary material continued

| **Study reference** | **Intervention (n = number of participants)** | **Study design** | **Number of CBT sessions and duration of CBT** | **Participants** | **Baseline predictor measure** | | **Clinical outcome measure** | **Result** |
| --- | --- | --- | --- | --- | --- | --- | --- | --- |
| Penades et al. (2010) | 1. CBTp (n=60)  2. SC (n=20) | Cohort study | Twenty-five sessions | Patients with schizophrenia (mean age 36 years) | | Measures of verbal IQ, working memory, psychomotor speed, verbal memory, non-verbal memory and executive function | A reliable improvement (an index value ≥1.96 above norm) in PANSS positive symptoms; pre- to post-treatment symptom change | ‘Improvers’ had better verbal memory than non-improvers at baseline; better logical memory at baseline predicted greater improvement in symptoms. |

BAI: Beck Anxiety Inventory, BCI: Beck Cognitive Insight, BDI: Beck Depression Inventory, BHS: Beck Hopelessness Scale, BIS: Birchwood Insight Scale, BPRS: Brief Psychiatric Symptoms Ratings Scale, CBT: Cognitive behaviour therapy, CPRS: Comprehensive Psychopathological Rating Scale, HoNOS: Health of the Nation Outcome scale, HVLT: Hopkins verbal learning test, ILSS: Independent Living Skills Survey, MADRS: Montgomery Asberg depression rating scale, MADS: Maudsley Assessment of Delusion Schedule, NART IQ: National Adult Reading test predicted IQ, PSYRATS: Psychiatric Symptom Rating Scale, RCT: Randomised controlled trial, SC: standard care.
